# Resistin expression in human monocytes is controlled by two linked promoter SNPs mediating NFKB p50/p50 binding and C-methylation

**DOI:** 10.1038/s41598-019-51592-0

**Published:** 2019-10-23

**Authors:** Dilip Kumar, Bernett Lee, Kia Joo Puan, Wendy Lee, Boris San Luis, Nurhashikin Yusof, Anand Kumar Andiappan, Ricardo Del Rosario, Jeremie Poschmann, Pavanish Kumar, Gennaro DeLibero, Amit Singhal, Shyam Prabhakar, Wang De Yun, Michael Poidinger, Olaf Rötzschke

**Affiliations:** 1 Singapore Immunology Network (SIgN), A*STAR (Agency for Science, Technology and Research), Singapore, Singapore; 2 Department of Otolaryngology, National University of Singapore, Singapore, Singapore; 3 Genome Institute of Singapore (GIS), Agency for Science, Technology and Research of Singapore (A*STAR), Singapore, Singapore; 4 Department of Biomedicine, University of Basel, Basel, Switzerland; 5 Stanley Center for Psychiatric Research, Broad Institute of MIT and Harvard, 75 Ames St., Cambridge, MA 02142 USA; 6 Centre de Recherche en Transplantation et Immunologie, Université de Nantes, Nantes, France

**Keywords:** Genetics, Gene regulation

## Abstract

Resistin is a key cytokine associated with metabolic and inflammatory diseases. Especially in East Asian populations, the expression levels are strongly influenced by genetic polymorphisms. Mechanisms and functional implications of this genetic control are still unknown. By employing reporter assays, EMSA, inhibition studies, bisulphite sequencing, ChIP-Seq and gene-editing we show that the p50/p50 homodimer known to act as repressor for a number of pro-inflammatory genes plays a central role in the genetic regulation of resistin in monocytes along with promoter methylation. In the common RETN haplotype p50/p50 constitutively dampens the expression by binding to the promoter. In an Asian haplotype variant however this interaction is disrupted by the A allele of rs3219175. The SNP is in very close linkage to rs34861192, a CpG SNP, located 280 bp upstream which provides an allele-specific C-methylation site. rs34861192 is located in a 100 bp region found to be methylated in the common but not in the Asian haplotype, resulting in the latter having a higher basal expression, which also associates with elevated histone acetylation (H3K27ac). Genotype associations within cohort data of 200 East Asian individuals revealed significant associations between this haplotype and the plasma levels of factors such as TGF-b, S100B, sRAGE and IL-8 as well as with myeloid DC counts. Thus, the common RETN haplotype is tightly regulated by the epigenetic mechanism linked to p50/p50-binding. This control is lost in the Asian haplotype, which may have evolved to balance the antagonistic RETN effects on pathogen protection vs. metabolic and inflammatory disease induction.

## Introduction

Resistin (RETN) was named for its ability to confer resistance to insulin^1^. In mice the main source of RETN is white adipose tissue but in humans it is primarily produced by cell populations other than adipocytes such as monocytes, macrophages and bone marrow cells^2^. As member of the adipokine family of cytokines, it modulates the release and effect of a diverse set of chemokines and cytokines by engaging an unknown receptor^3,4^. RETN has been widely studied in the context of diseases.

The plasma concentration of RETN is reportedly increased in obesity, diabetic, cardiovascular and inflammatory conditions, as reported in several genetic studies^4–6^. Multiple SNPs had been identified that associate with either RETN mRNA or plasma protein levels and disease manifestations^7–10^. In Asian populations RETN expression was found to be strongly regulated by two closely linked SNPs, rs3219175G/A and rs34861192G/A, where plasma levels where positively associated with the A alleles of the two SNPs^7,8,10^. The contribution of rs1862513, due to its partial linkage to the former, is still a matter of debate^7,9–11^. Compared to the ancestral G-G haplotype of rs3219175-rs34861192, the derived A-A haplotype has been associated with high resistin expression^7^. Higher plasma levels have also been associated with the methylation state of cg02346997, a CpG residue in the promoter region of the gene^12^, and the allele-dependent methylation of the CpG-SNP rs1862513^12,13^. However, at least in Asian populations rs34861192-rs3219175 still seems to be the most significant genetic determinant for RETN expression^7–9,12^.

Mechanistic studies in adipocyte and monocytic cell lines suggested that the transcriptional regulation appears to be mediated by factors such as SP1/SP3, ADD1/ SREBP1c and C/EBPα^14–16^. Little is known however about the genetic control of RETN in monocytes, the main producers of RETN in humans^7,12^. Here we show that in east Asian populations the allele-specific expression of RETN in monocytes is facilitated by tight interplay of genetic and epigenetic mechanisms. With methods such as promoter Luciferase assay, EMSA, p50 peptide inhibition, CRISPR knock out of NFKB1/p50, bisulfite DNA sequencing and genome-wide eQTL-analysis we show that rs3219175 controls the binding of NFkB1 p50/p50 homodimer, a well-established transcriptional repressor of pro-inflammatory genes. Binding of the factor to the common RETN haplotype is associated with a the C-methylation of a narrow promoter region with rs34861192 acting as allele-specific methylation site. All of these interactions are lost in the (less frequent) Asian haplotype, where the higher basal expression of RETN is associated with extensive histone acetylation (H3K27ac). Genotype correlations with the cohort data of 200 ethnic Chinese individuals confirmed the expected influence of the two SNPs on the RETN plasma concentration but also revealed nominally significant associations (p value > 0.05) with cytokines and blood cell levels linked to RETN related disease manifestations.

## Results

### Resistin eQTL

**Figure 1:**
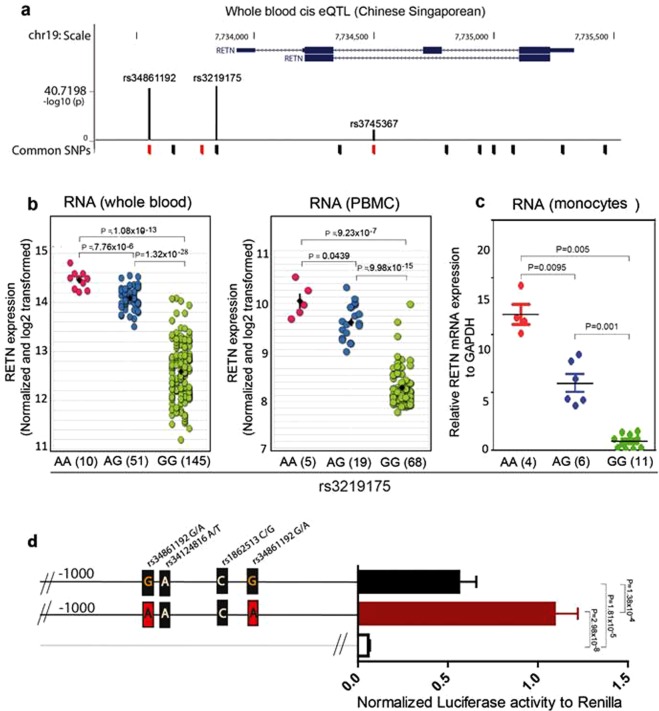
Resistin expression is regulated by rs34861192 and rs3219175. (**a**) Resistin eQTL in whole blood samples. The panel shows the location of eQTL-SNPs within in the resistin (RETN) gene locus. The position of common SNPs relative to the RETN gene is indicated on the x-axis (CpG-SNPs are in red); the -log10 (p value) indicated on the y-axis refers to their association with RETN-mRNA detected in 202 whole blood samples of our Singapore-Chinese cohort^19^. (**b**) rs3219175 genotype-associations. The levels of RETN mRNA isolated from whole blood cells (left panel), PBMC samples (right panel) of the Singapore-Chinese cohort were plotted against the genotypes of the RETN promoter SNP rs3219175. The mRNA expression displayed has been normalized and log2-transformed; p values were calculated using Mann-Whitney unpaired test. (**c**) Genotype-association in monocytes. The correlation with rs3219175 is shown for monocytes isolated from 21 individuals of Chinese ethnicity. The mRNA expression displayed on the y-axis represents the level of RETN mRNA expression in reference to GAPDH. (**d**) Reporter assay. The bar chart shows the result of a Luciferase reporter assay for the RETN promoter, in which the G-G and the A-A haplotype (rs34861192-rs3219175) were compared. The experiments were carried out in U937 cells, the promoter-driven firefly luminescence was normalized by renilla luminescence. The baseline signal (empty bar) was derived from a luciferase vector lacking the promoter fragment (PGL3-BASIC).

A genome-wide eQTL analysis of whole blood samples from 206 of donors of Chinese ethnicity^17–20^ with more than 2 million SNPs where SNPs within 100,000 bp were associated with the mRNA expression was conducted. The analysis revealed a significant association with 4 cis SNPs with RETN mRNA (Fig. 1a and Table 1). Among the cis SNPs all 4 had adjusted p values < 1 × 10^−04^ (rs3219175: 1.91 × 10^−41^, rs34861192: 3.16 × 10^−38^, rs10401670: 1.77 × 10^−05^ and rs32745367: 5.19 × 10^−05^). rs3219175 and rs34861192 were located 358 and 638 bp upstream of the TSS with nearly perfect linkage to each other (r^2^ > 0.99). Although the upstream promoter SNP rs10401670 and intronic SNP rs32745367 was linked to the pair with an r^2^ value of only 0.4361and 0.4367 respectively (Table 1), the association with the RETN expression was completely lost when performing a PLINK analysis (with rs3219175 as conditional SNP). Thus, only rs3219175-rs34861192 appear to affect RETN expression (Supplementary Table 1).

**Table 1:** Whole blood eQTL analysis.

CHR	SNP ID	Position	Allele 1	n	Regression coefficient	Standard error	t statistic	p	FDR	Distance from TSS	r2
19	rs3219175	7733855	A	206	1.223	0.06578	18.6	8.307E-46	1.90634E-41	−358	1
19	rs34861192	7733575	A	199	1.237	0.06807	18.17	5.592E-44	3.16325E-38	−638	>0.99
19	rs10401670	7742802	T	206	0.4577	0.08014	5.711	3.928E-08	1.76759E-05	8589	0.44
19	rs3745367	7734511	A	206	0.4461	0.0815	5.474	1.283E-07	5.19339E-05	298	0.44

The table indicates all cis SNPs (with a window of 100,000 bp around RETN gene locus) associated with resistin mRNA expression (FDR < 0.05), SNP id with their respective genomic position within chromosome 19, allele 1 (minor allele), the number of samples (n), regression coefficient, standard error, t statistic and the nominal and adjusted p values, as well as the location with regard to RETN gene locus, the linkage disequilibrium (r2) with rs3219175, and the distance from the transcriptional start site (TSS) for all significant SNPs.

The plot of the genotypes of rs3219175 *vs*. the RETN mRNA level of whole blood samples indicates that high expression is associated with the A allele of the SNP (Fig. 1b, left panel); a clear increase was observed from GG to AG (p = 1.32 × 10^−28^) and from AG to AA (p = 7.76 × 10^−06^). A very similar association was also evident when the correlation was carried out with PBMC samples of the cohort (p = 9.98 × 10^−15^ and p = 4.39 × 10^−2^, respectively) (Fig. 1b left panel). As monocytes were the likely source for the RETN mRNA, we repeated the experiment with monocytes isolated from PBMC of 21 healthy volunteers of Chinese ethnicity (Fig. 1c). The correlation of rs3219175 with RETN mRNA levels (GG/AG: p = 0.001; AG/AA: p = 0.0095) showed the same trend as observed with whole blood and PBMC but also it revealed that these cells are particularly affected by the polymorphism: the fold change from GG → AG and GG → AA was 13.1 and 30.4, respectively. The strong association with the SNP pair was also evident in the published eQTL data by Raj *et al*.^21^. The studies were based on large cohorts of more than 100 individuals where rs3219175 and rs34861192 associated with p values < 1 × 10^−14^ in both East Asian and American African populations (Supplementary Table 2).

Due to the close linkage between rs3219175 and rs34861192 only two haplotypes exist for the two SNPs: the common G-G haplotype and the less frequent A-A haplotype, present in Est Asian populations with an allele frequency of about 0.2. Based on the eQTL analysis the latter appears to be associated with a more than 30-fold higher baseline expression of the RETN gene. To formally demonstrate the impact of these two haplotypes on transcription we therefore analyzed the haplotype-associated expression driven by the RETN promoter in a luciferase reporter assay (Fig. 1d). Matching fragments of the RETN gene promoter (1 kB upstream to ATG start codon) from a G-G and a A-A donor (rs34861192-rs3219175) were cloned into a luciferase vector system and transferred into the monocytic cell line U937 (Supplementary Fig. 1). Sequencing of the fragments revealed that, besides the two SNPs, an allelic difference was detected only for rs1862513. While the latter had been associated with RETN expression and T2D^8,11,22^, no significant effect was observed in the reporter assay when the alleles of this SNP were swapped by site directed mutagenesis (Supplementary Fig. 2) which is also consistent with an earlier cohort report showing rs34861192-rs3219175 haplotype as predominant genetic regulator of resistin^7^. More importantly, in line with our QTL data, the promoter luciferase assay showed a substantially stronger signal for the A-A haplotype compared to the G-G haplotype (Fig. 1d).

### rs3219175 controls the binding of NFKB1 (p50/p50) homodimer

**Figure 2:**
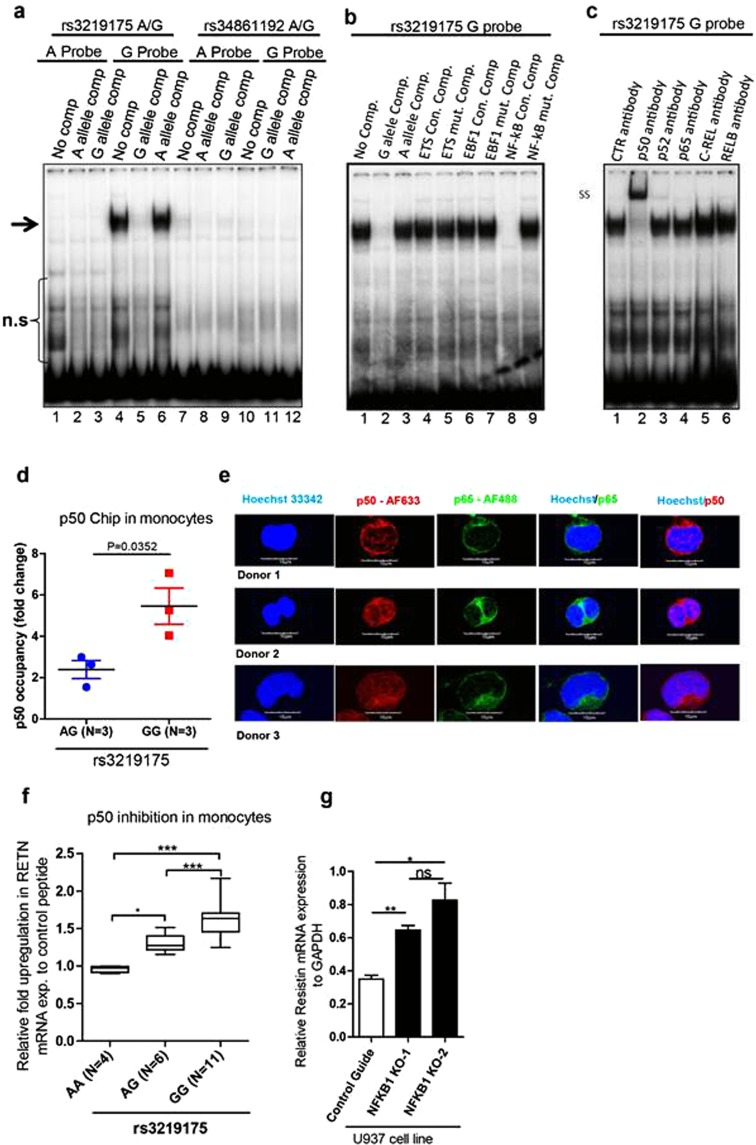
rs3219175 regulates the binding of NFkB1 p50/p50 homodimers. (**a**) Transcription factor binding to RETN SNPs. EMSA experiments with nuclear extracts from primary monocytes were carried out with radiolabeled probes containing the A allele (lane 1–3) or the G allele of rs3219175 (lane 4–6), and with probes containing the A allele (lane 7–9) or the G allele of rs34861192 (lane 10–12). The binding was competed with an excess of unlabeled oligonucleotides of the A and G allele of the respective SNP as indicated. The specific bands are marked by an arrow, n.s. indicates non- specific interactions. (**b**) Competition with consensus motifs. The binding to the radiolabeled rs3219175 G probe was competed by an excess of unlabeled oligonucleotides containing either the consensus sequence or a mutated version of ETS1 (lane 4, 5), EBF1 (lane 6, 7) and NF-κB family (lane 8, 9). As control, the competition was also carried out with the unlabeled rs3219175 G (lane 2) and A probe (lane 3). (**c**) Super-shift assay. Super-shift assays were carried out with the radiolabeled rs3219175 G probe and antibodies against NF-κB p50 (lane 2), p52 (lane 3), p65 (lane 4), c-REL (lane 5), REL-B (lane 6) or a control antibody (lanes 1). Super-shifted bands (SS) and bands representing NF-κB p50 (p50) are indicated; selectivity and functionality had been validated for all antibodies in supershift assays (Supplementary Files 7 and File 8). (**d**) NF-κB p50-ChIP assay. A ChIP assay with nuclear samples from monocytes of 3 AG- and 3-GG donors (rs3219175) was carried out with p50-specific antibodies or isotype-matched control antibodies. The p50 occupancy, depicted as fold change compared to the isotype, was determined by qPCR amplification of the respective region in the RETN promoter. (**e**) Constitutive localization of p50 in the nucleus of monocytes. Images obtained by confocal microscopy of non-activated monocytes from three different donors are shown. The images display the nucleus staining (Hoechst 33342), NF-κB p50 (p50-AF633), NF-κB p65 (p65-AF488), as well as the overlays Hoechst/p65 and Hoechst/p50. (**f**) p50 peptide-inhibition assay. Freshly isolated monocytes from donors genotyped for rs3219175 (AA = 4, AG = 6 and GG = 11) were treated with 30 µM of either p50 specific peptide inhibitor (NF-κB SN50) or control peptide for 24 h. The box plots indicate the relative upregulation in RETN mRNA expression in the presence of the inhibitor in reference to the peptide control. (**g**) NFKB1 p50 knock out in U937 cells. The monocytic cell line U937 used for these experiments is homozygous for the p50/p50-sensitive G-G haplotype (rs3219175-rs34861192). The RETN-mRNA expression is shown for two independent U937 clones, in which NFKB1 was knocked out by CRISPR (NFKB1 KO-1 and NFKB1 KO-2), as well as for a clone treated with a control guide RNA.

In order to determine, if the binding of any transcription factors to the RETN promotor is regulated by of the two SNPs, we carried out electrophoretic mobility shift assays (EMSA) with nuclear extracts from freshly isolated monocytes. While no specific binding was detected for the probe containing the rs34861192 SNP, some nuclear factor(s) clearly associated to the rs3219175 probe in an allele-specific way (Fig. 2a, Supplementary Table 3 and Supplementary Fig. 3). A distinct band was detected for the G allele but the A allele of the SNP. The specificity of the binding was confirmed by competition with an excess of unlabeled probes. Only the self-competition with unlabeled G probes prevented the binding to the radiolabeled probe, while a 100 fold excess of the unlabeled A probe had no effect (Fig. 2a).

In order to identify candidates for the allele-specific binding to the rs3219175 site, we analyzed the sequence of the G- and A-probe by the web-based TRANSFAC tool (http://gene-regulation.com/pub/databases.html). Only three candidates, ETS, NFkB and EBF1, were predicted to bind preferentially to the G probe (Supplementary Table 4). Competition with unlabeled probes representing the consensus sequence for the binding of these factors indicated that only the probe for NFkB family members blocked the formation of the band (Fig. 2b, Supplementary Table 3 and Supplementary Fig. 3). The inhibition was as strong as observed for the unlabeled G probe, while no interference was detected for ETS and EBF1 consensus probes. Competition by the consensus probe for NFkB was abrogated when the binding motif was destroyed by exchanges in crucial base pairs (Fig. 2b). Similar results were obtained when radiolabeled NFkB-probes were competed with unlabeled G-probes of rs3219175 (Supplementary Fig. 4).

To identify the specific NFkB family member binding to the RETN promotor, supershift assays were performed with antibodies specific for p50, p52, p65, REL-B and c-REL. For each antibody the specificity and function was confirmed in EMSA supershift/blocking experiments with radiolabeled probes of the respective consensus motifs (Supplementary Fig. 5). When using the rs3219175 G probe a supershifted band was observed only with the p50-specific antibody (Fig. 2c and Supplementary Fig. 4). None of the other antibodies blocked or supershifted the band, indicating that p50/p50 homodimers are binding to the site.

In order to assure that the allele-specific interaction of p50 with rs3219175 occurs also naturally we carried out ChIP assays with monocytes isolated from six genotyped donors (Fig. 2d). Genomic DNA fragments from three heterozygous AG and three homozygous GG donors were precipitated with p50-specific antibodies and analyzed by qPCR. The primer pair covered a region of the *RETN* promoter that included the rs3219175 SNP (Supplementary Fig. 1). In line with the results from the EMSA and supershift experiments, a significant increase in the amount of *RETN* promoter DNA was detected in the p50 precipitate from GG donors compared to AG donors (Fig. 2d). Thus, in human monocytes, p50 is associated in an allele-specific way with rs3219175 G.

In non-activated cells, NFkB family members typically reside in the cytosol^23^. One exception is the p50/p50 homodimer, as a fraction of this complex is constitutively present in the nucleus^24,25^. This was confirmed also for our freshly isolated monocytes where immunofluorescence confocal microscopy with a p50- and p65-specific antibody; p50 specific staining was clearly evident inside the nucleus, whereas p65, which is tightly regulated by IkB alpha, was virtually absent from this compartment (Fig. 2e). Within the nucleus, p50/p50 homodimers act as transcriptional repressors^26,27^. Functional inactivation with p50-specific peptide inhibitors confirmed that the same applies also for RETN. When freshly isolated monocytes were incubated with the inhibitor a significant upregulation of RETN mRNA expression was detected. In line with the allele-specific binding observed in EMSA ChIP experiments, the effect was observed only in cells of the rs3219175 GG genotype but not for AA genotype (Fig. 2f). A similar reversion of the inhibitory effect was also observed when NFKB1/p50 was knocked out in the monocytic cell line by CRISPR/CAS9. A significant upregulation in RETN mRNA expression was observed in two independently generated U937 −/− clones (Fig. 2g and Supplementary Fig. 6).

### The rs34861192-rs3219175 haplotype controls promoter methylation

As mentioned above, the CpG SNP rs34861192 is closely linked with rs3219175 (r^2^ > 0.99). In contrast to the latter, it did not show any allele-specific binding to nuclear factors (compare Fig. 2a) but could potentially contribute to the gene regulation by providing an allele-specific site for C-methylation. The same may also apply for rs1862513, another CpG SNP partially linked to the pair (r^2^ = > 0.47)^7,11,22^, whose methylation state is reportedly associated with resistin expression^12,13^. Likewise, also the methylation of cg02346997, a non-polymorphic CpG site in the immediate promoter region of the gene, has been directly associated the resistin expression^12^.

**Figure 3:**
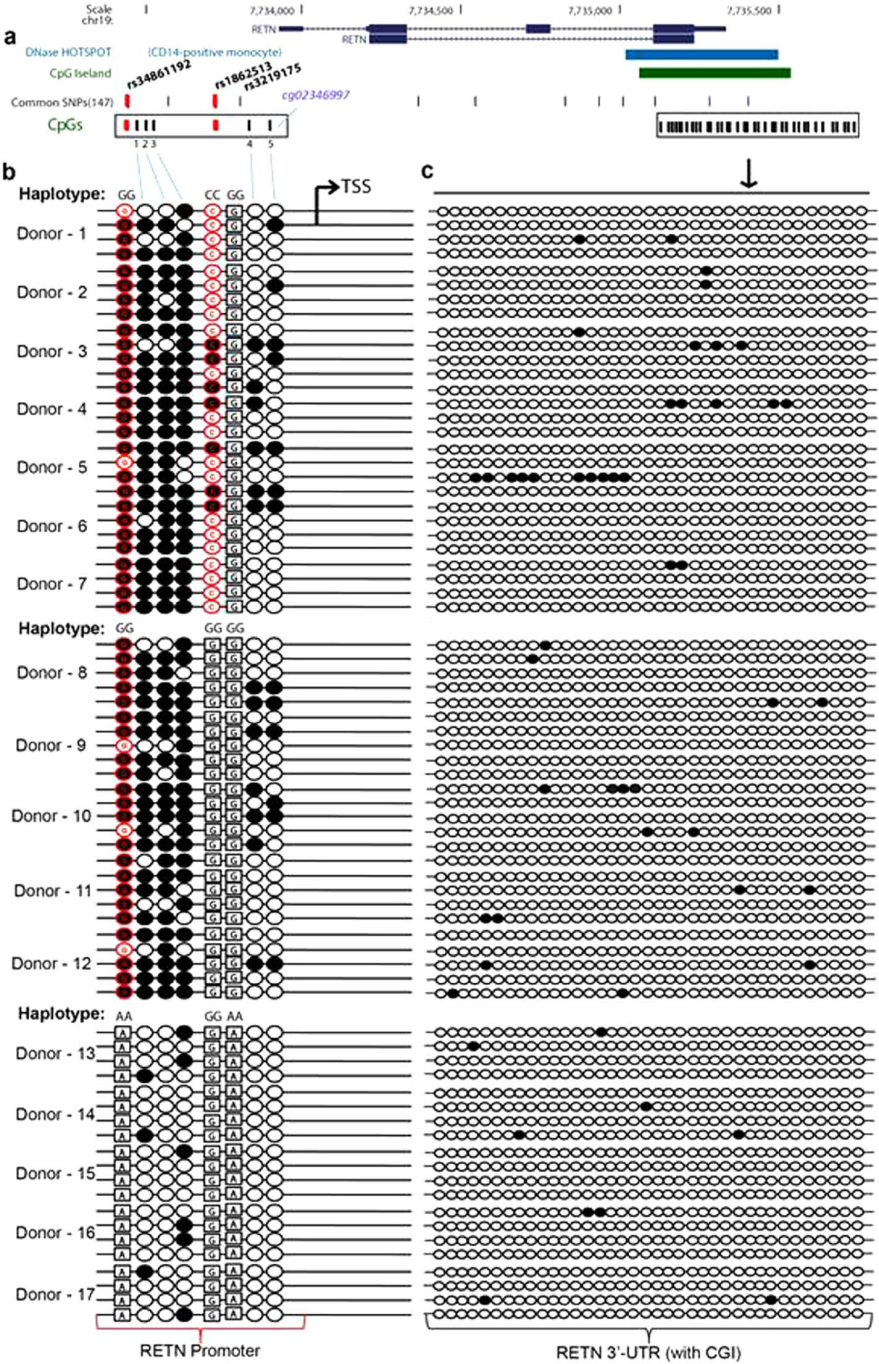
Allele-dependent C-methylation of the RETN promoter. (**a**) Schematic overview of the RETN gene locus. The figure depicts the intron/exon structure of RETN, together with the location of a monocyte-specific DNase hotspot (light blue track), a CpG island (green track), common SNPs and CpG pairs (CpG-SNPs are indicated in red and five non-polymorphic CpGs are indicated as 1, 2, 3, 4 and 5 in black, with CpG_5 also by its illumina loci identifier cg02346997). The location of rs34861192, rs1862513 and rs3219175 is indicated. Regions covering CpGs in the promoter and 3-UTR that were analyzed by bisufite sequencing are framed by box; the DNase hotspot was obtained from UCSC genome browser (http://genome.ucsc.edu). (**b**) C-methylation marks in the promoter. The 470 bp RETN promoter region analyzed by bisulfite sequencing contained 7 CpG pairs (including two CpG created by G alleles of rs34861192 and rs1862513). For each donor the methylation state of four independently sequenced clones is shown. All donors were homozygous for rs34861192 and rs3219175 with 7 donors having the G-C-G (Donors 1–7), 5 donors having the G-G-G (Donors 8–12) and 5 donors the A-C-A haplotype (Donors 13–17). The methylation state of each CpG element is represented by circles (full circle: methylated, empty circle: demethylated); boxed alleles refer to the non-CpG state of a SNP; the location of the TSS as well as of rs34861192, rs1862513 and rs3219175 are indicated. (**c**) C-methylation in the 3′ CpG island. The methylation state is shown for a 495 bp region in the 3′-UTR region containing 37 CpG pairs.

In order to determine the allele-specific methylation pattern of the RETN promoter in monocytes, we therefore carried out a bisulfite sequencing-analysis of monocyte DNA isolated from donors of the rs34861192- rs1862513- rs3219175 haplotypes G-C-G (12 donors) G-G-G (5 donors) and A-G-A (5 donors). The C-methylation analysis covered a 470 bp segment located 301 bp upstream of the transcriptional start site (TSS) of RETN. The segment contained 7 CpG pairs including cg02346997 as well as the two CpG SNPs formed by the C alleles of rs34861192 (counter strand) and rs1862513 (Fig. 3a). As a reference, we also analyzed a 500 bp segment of the 3′ RETN UTR containing a prominent CpG island (Fig. 3a, Supplementary Fig. 7).

Within all sequenced human subpopulations the G alleles of rs34861192 and rs3219175 are dominant. As these alleles are associated with low basal RETN expression (compare Fig. 1), it was assumed that promoter or other regulatory gene elements in the respective haplotypes display a high level of C-methylation. In line with this assumption, in nearly all of the analyzed monocyte samples of the G-C-G and G-G-G haplotype, we found that the C-allele on the counter-strand of rs34861192 was methylated (Fig. 3b upper, middle panel and Supplementary Table 5). Moreover, in these haplotypes the methylation was not restricted to rs34861192 alone but extended 70 bp downstream of the SNP covering three additional CpG pairs (termed here CpG_1, CpG_2 and CpG_3) (Fig. 3a,b upper, middle panel and Supplementary Table 5). All remaining CpGs were mostly un-methylated, which also included CpG_4 (cg02346997), the CpG pair whose methylation state was reported to be associated with RETN expression levels^12^.

The two CpG SNPs rs34861192 and rs1862513 exhibited a striking difference in their degree of methylation when in the allelic state forming the CpG site. While rs34861192 shows a high level of methylation (~90%) rs1862513 is only weakly methylated (18.8%) (Supplementary Table 5). The state of methylation associated with the rs34861192 genotype extended to the entire promotor region. The methylation analysis of 5 additional CpG sites revealed and average of 60.4% and 54.8% methylation for the G-C-G and G-G-G haplotypes of rs34861192-rs1862513-rs3219175 haplotypes and only 6.4% for A-G-A (Supplementary Table 5, Supplementary Fig. 8). This analysis was based on 93 independent DNA clones from monocytes isolated from 22 genotyped donors (Supplementary Table 5). Thus at least in primary human monocytes promoter methylation seems to be controlled by rs34861192 while rs1862513 seems to play only a minor role in this process. This result appears to be in contrast to a prior report^13^ reporting a correlation of the methylation of this SNP with plasma resistin levels. However, we cannot rule out that in other populations with LD <1 (rs34861192/rs3219175) this SNP might have a different impact.

In contrast to the common G-C-G and G-G-G haplotypes, A-G-A is virtually absent in Caucasian and South Asian populations. Only in East Asians it is present with an allele frequency of 0.20^28^. Consistent with the high RETN expression associated with the A alleles of rs34861192 and rs3219175 (compare Fig. 1), promoter methylation was almost absent in all samples of the A-G-A haplotype (Fig. 3b, lower panel). This applied not only to rs34861192, which in this allelic state has lost its methyl-acceptor function, but also to the three adjacent CpG pairs CpG_1, CpG_2, CpG_3. Independent of allelic state of the SNP, the CpG Island at 3′ UTR was always almost completely demethylated Fig. 3c. In contrast to the promoter, it is apparently not directly involved in the genetic control of the gene (Fig. 3b,c, right panel).

### Haplotype-dependent H3K27 acetylation

The bisulfite sequencing analysis revealed that promoter methylation is restricted to a narrow region proximal to the rs34861192 CpG SNP. The methylation of this region is closely linked with the binding of p50/p50 homodimer to rs3219175 located about 250 bp downstream of this site. While p50/50 homodimer does not seem to directly activate DNA-methylases, it has been reported to suppress target genes through recruitment of histone deacetylases^27,29^. C-methylation is typically inversely correlated with histone-acetylation^29,30^. To determine the effect of the rs3219175-rs34861192 haplotype on the H3 K27 acetylation (H3K27ac), we therefore carried out histone acetylation QTL (haQTL) analysis with monocytes isolated from 32 donors of Asian ethnicity. As part of a genome-wide approach to identify haQTLs, the reads from the H3K27ac ChIP-seq were used directly to identify SNPs and to infer genotype likelihoods.

**Figure 4:**
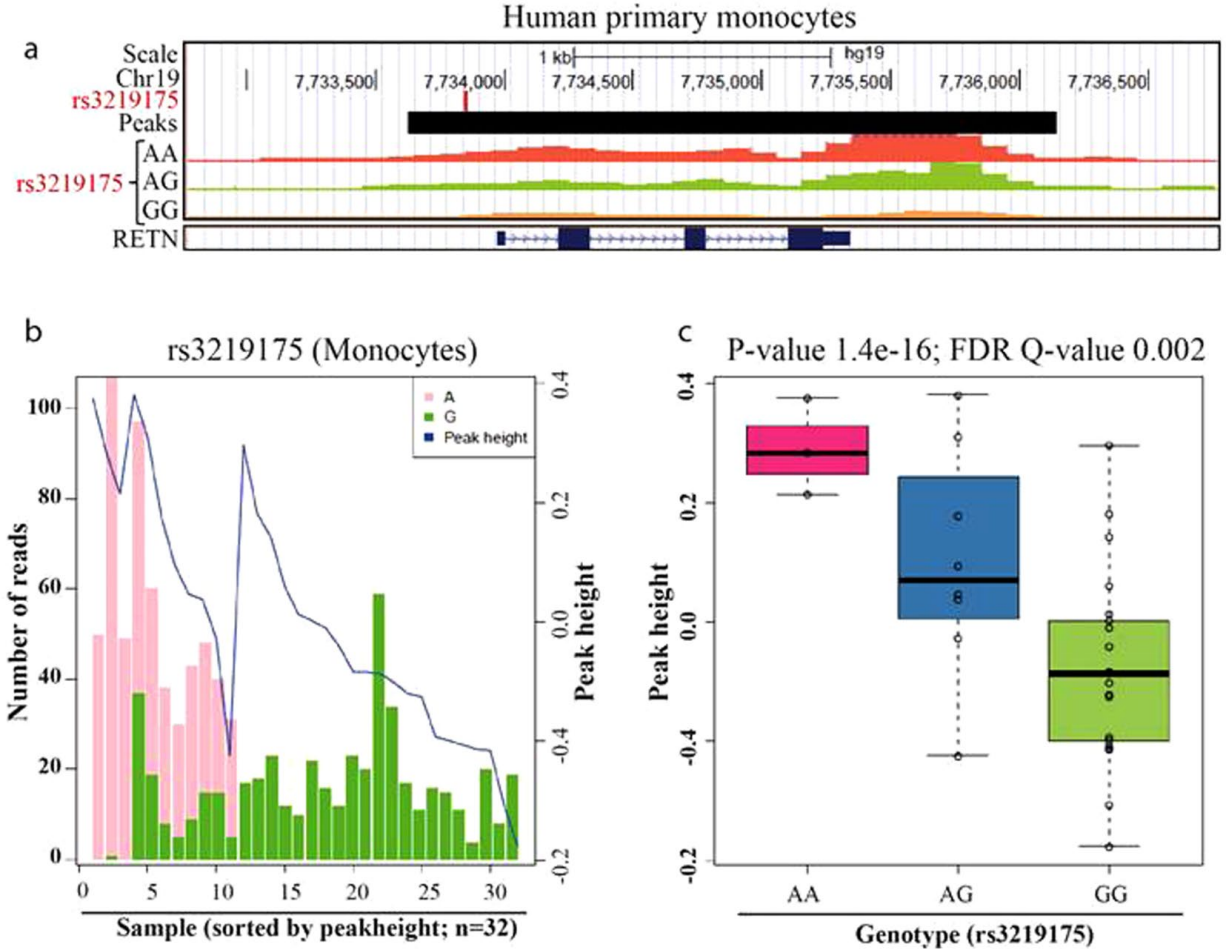
Haplotype dependent acetylation of the RETN gene locus. (**a**) Genome browser view of the RETN gene locus. The figure depicts the RETN region analyzed for H3K27 acetylation, in reference to the location of rs3219175. Representative H3K27me3 ChIP-seq tracks for monocytes from individuals of the three rs3219175 genotypes (AA, AG and GG) are shown. All acetylation tracks are plotted on the same fold-enrichment scale. (**b**) Read depth analysis. The bar chart displays the number of sequencing reads covering the A allele (pink) and the G allele (green) of rs3219175 within 32 individuals. The blue line represent peak heights of the peaks for each genotype (**c**) Peak height association. The boxplots indicate the height of the H3K27ac peak in reference to the rs3219175 genotype.

Within the 32 Chinese individuals we identified 21 GG, 8 AG and 3 AA genotypes of rs3219175. The anlysis of the H3K27ac ChIP-seq reads for these genotypes revealed that rs3219175 is located within a nearly 2 kb H3K27ac peak covering the complete RETN gene (Fig. 4a). Genome browser tracks from 3 representative individuals further showed that the acetylated peak heights vary between the genotypes, gradually falling from AA over AG to GG (Fig. 4a). Consequently, the analysis of the number of reads covering the G and A allele in these 32 individuals showed an enrichment of the A allele, suggesting allelic imbalance of the H3K27ac ChIP-seq signal in favor of the A allele (Fig. 4b). In the complete set of individuals, a significant (p value = 1.4 × 10^−16^ and FDR Q value 0.002) genotype-dependent H3K27ac peak height changes appeared in concordance with gene expression, which is highest for AA and lowest for GG (Fig. 4c) (Genome wide haQTL data is in submission; Poschmann & del Rosario *et al*.)^31^.

### The allelic state of rs3219175-rs34861192 is associated with variations in cytokine levels of resistin-related pathways and mDC frequency

The protein levels of RETN in the plasma had been studied in the context of different diseases^3^. Besides correlations with clinical parameters^32–35^, RETN protein levels also appeared to modulate several chemokines and cytokines^3^. To analyze the impact of the RETN haplotypes in an East Asian population, we used the data of our cohort of 202 individuals of Chinese ethnicity. In addition to the genotype information, quantitative data on plasma components was available that covered more than 100 different cytokines, chemokines and hormones, including RETN^18^.

**Figure 5:**
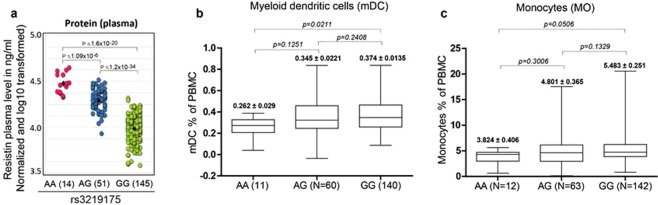
Correlation of rs3219175 with plasma proteins and myeloid cell counts. (**a**) Resistin plasma protein. The three genotype states of RETN SNP rs3219175 (AA, AG and GG) were correlated with the plasma protein concentration of resistin using data collected for the Singapore-Chinese cohort^18,19^. The y-axis represents the log10-transformed normalized plasma concentration (ng/ml), p value was calculated by using a linear regression model (matrix eQTL). Association with plasma proteins. (**b**,**c**) Association of rs3219175 with the myeloid cell count. The association of rs3219175 with the cell counts of mDC (middle panel) and monocytes (right panel) is shown. The whole blood samples of the Singapore-Chinese cohort were analyzed by FACS. The cell counts of the population defined by gating (Supplementary Fig. 9) are expressed as percentage of total PBMC. The average percentage of the cells as well as sample numbers for each genotype combination are indicated; statistical analysis was performed using Mann-Whitney unpaired t-test.

As expected, the direct correlation of the RETN plasma concentration with the genotypes of rs3219175 revealed a strong association. The protein levels in the plasma significantly increased from GG to AG (p = 1.27 × 10^−34^) and from AG to AA (p = 1.09 × 10^−6^) (Fig. 5a and Table 2). Overall, more than 3-fold higher RETN levels were observed in AA individuals compared to GG individuals. An additional 11 cytokines correlated with nominal p values (p < 0.05) in an apparent trans-association with the rs3219175 genotype (Table 2). The association was lost however, on multiple testing. The list included a number of inflammation-associated factors such as TGF-β2, TGF-β1, S100B, MCP-2, sRAGE and IL8, of which only sRAGE was positively associated with the high expressing AA genotype, while all other factors were inversely associated. When using this list for an Ingenuity Pathway Analysis (IPA), “HMGB1 pathway” (p = 9.26 × 10^−10^) followed by “Hepatic fibrosis/Stellate cell activation” (2.68 × 10^−09^) was suggested as the two top canonical pathways associated with the RETN polymorphism (Table 3). Similar to RETN, HMGB1 has been associated with T2D, CAD and infectious diseases^36–38^. Hepatic fibrosis is well known to be mediated by the RETN pathway^39,40^. The top-ranked category of ‘diseases and bio functions’ was “inflammatory response” (p value 3.84 × 10^−2^–6.78 × 10^−7^), which is concordant with the role of RETN-in inflammatory related pathways.

**Table 2:** The associations of RETN SNP rs3219175 with plasma biomarkers.

Analytes	Position	Allele 1	n	Regression coefficient	Standard error	t statistic	p	AA genotype	GG genotype
Resistin	7733855	A	197	2.76E-01	0.01641	16.79	1.154E-39	4.476 (±0.058)	3.984 (±0.024)
TGFB2	7733855	A	197	−0.04273	0.01475	−2.896	0.004207	2.486 (±0.049)	2.569 (±0.019)
TGFB1	7733855	A	187	−0.04649	0.01843	−2.522	0.01252	2.918 (±0.056)	2.963 (±0.027)
S100-B	7733855	A	92	−0.1348	0.0553	−2.437	0.01681	0.883 (±0.547)	1.198 (±0.065)
MCP-2	7733855	A	187	−0.03991	0.01673	−2.385	0.01809	1.270 (±0.0.052)	1.309 (±0.024)
IL-8	7733855	A	179	−0.1777	0.07641	−2.326	0.02118	2.858 (±0.048)	2.881 (±0.017)
sTNFRI	7733855	A	197	−0.03211	0.01412	−2.274	0.02405	0.311 (±0.397)	0.526 (±0.089)
sRAGE	7733855	A	198	0.07566	0.0346	2.187	0.02996	2.087 (±0.055)	1.991 (±0.053)
sTNFRII	7733855	A	197	−0.02976	0.01448	−2.055	0.04119	3.554 (±0.072)	3.571 (±0.020)
sVEGFR2	7733855	A	197	−0.02469	0.01205	−2.049	0.04181	4.015 (±0.061)	4.071 (±0.016)
MIG	7733855	A	198	−0.06777	0.03331	−2.035	0.04325	2.553 (±0.147)	2.601 (±0.050)

The table summarizes the association data of plasma proteins correlating with rs3219175 (genomic position within chr19: 7733855) with nominal p values < 0.05. For each analyte the allele 1 (minor allele), the number of samples (n), regression coefficient, standard error, t statistic and the nominal p value as well as the average plasma concentration in individuals of the AA and GG genotype are listed.

**Table 3:** Summary of IPA analysis based on rs3219175 associated plasma analytes.

Top Canonical pathways	p value	Overlapping genes	Genes
**HMGB1 pathway**	**9.26E-10**	**4.2% 5/118**	AGER, TGFB1, TGFB2, TNFRSF1A, TNFRSF1B
Hepatic Fibrosis/Stellate Cell Activation	3.68E-09	3.2% 5/155	VEGFR2, TGFB1, TGFB2, TNFRSF1A, TNFRSF1B
p38 MAPK Signaling	9.78E-08	4.0% 4/101	TGFB1, TGFB2, TNFRSF1A, TNFRSF1B
Hepatic Cholestasis	3.06E-07	3.0% 4/134	TGFB1, TGFB2, TNFRSF1A, TNFRSF1B
Tight Junction Signaling	3.25E-07	2.9% 4/136	TGFB1, TGFB2, TNFRSF1A, TNFRSF1B
**Top Diseases and Bio Functions**	**p-value**	**Molecules**	
**Inflammatory Response**	**3.84E-02**–**6.78E-07**	**4**	
Endocrine System Disorders	2.05E-03–8.13E-06	2	
Gastrointestinal Disease	2.05E-03–8.13E-06	2	
Metabolic Disease	2.05E-03–8.13E-06	2	

Ingenuity Pathway Analysis (IPA analysis): rs3219175 associated Chemokine and Cytokines.

Plasma Chemokines and Cytokines associated with rs3219175 (see Table 2) were analyzed using IPA analysis (www.qiagenbioinformatics.com) for the enrichments of associated genes in the plasma with known biological pathways, diseases and bio-functions.

Lastly, flow cytometry data of this cohort further revealed a significant increase of the percentage of myeloid dendritic cells (mDC) in the GG versus the AA genotype (p = 2.11 × 10^−2^) (Fig. 5b, Supplementary Fig. 9). A similar trend was also observed for monocytes (5.06 × 10^−2^) (Fig. 5c). The data is consistent with prior studies^41–46^ indicating an inverse correlation of myeloid cell counts with T2D, coronary heart disease and inflammatory diseases, all of which are positively associated with RETN expression.

## Discussion

In this study, we provide insight into the mechanism of genetic control of the RETN expression in human monocytes. It is facilitated by a pair of closely linked promoter SNPs (rs34861192 and rs3219175) that silence the gene in a concerted fashion via recruitment of the transcriptional repressor NFKB1-p50/p50-homodimer in conjunction with the allele specific C-methylation of the RETN promoter. The NFKB1 p50 subunit is an important regulator of NF-kB activity and, as p50/p50 homodimer, plays an important role in the suppression of pro-inflammatory genes. Transcriptional repression by this complex can be achieved in two ways: passively by competing for binding sites with other activating NF-kB complexes and actively by recruiting chromatin modifiers such as p50/p50:HDAC1 and p50/p50:EHMT1 promoting chromatin-condensation and -remodeling through histone modification^26,27,29,47^. While it is still unknown if NFKB complexes other than p50/p50 can bind to the RETN promotor, the active repression mechanism is strongly supported by the C methylation of the promoter associated with p50/p50 binding and hypo-acetylation of H3K27 throughout the entire gene body.

The binding of the p50/p50 homodimer to the promoter region is controlled by rs3219175. In EMSA and ChIP assays we demonstrated a specific interaction with the G allele of the SNP, and the RETN repression was reversed by a p50-specific peptide-inhibitor as well as by CRISPR-mediated knock out NFKB1. Moreover, with confocal microscopy we also showed that p50 is in fact constitutively present in the nuclear compartment of primary monocytes.

While rs3219175 controls the binding of p50/50, rs34861192 forms an allele-dependent site for C-methylation. Both the methylated CpG elements and p50/p50 homodimer provide interaction sites for methyl-CpG-binding proteins (MBPs) and histone deacetylases, which drive the local closing of the chromatin^29,48,49^. The coordination of the allele-specific suppressor mechanisms based on p50/p50 binding and C-methylation is ensured by the nearly perfect linkage of s3219175 with the rs34861192 CpG-SNP. However, the allele-specific C-methylation is not restricted to the CpG SNP but extends to three neighboring static CpG pairs, presumably to facilitate an effective closing of the promoter. Notably, the prominent CpG island located at the 3′ end of the gene is not involved in the process as its methylation state remains unchanged. The same applies also for cg02346997, a CpG site in the promotor previously reported to be associated with the expression of RETN in monocytes.

In contrast to a number of earlier reports^7,50,51^ we also did not observe any major impact of rs1862513. Neither the reporter assay nor the analysis of the haplotypes-specific methylation states revealed any major influence of this site in monocytes. In two of these prior studies^12,13^ the methylation of the CpG SNP was found to be inversely associated with plasma resistin levels. However, the SNP is linked with the dominant SNP rs34861192 with an r^2^ ranging from 0.47 to 0.57 in Asians^7,11,22^. As a CpG SNP its methylation is strongly correlated with its genotype (Supplementary Table 5). While a genetic association between rs1862513 and resistin levels was also reported for a Japanese cohort, in this study the association was completely lost when corrected for the influence by rs34861192^7^. Also a number of *in vitro* reporter studies also indicated an influence of rs1862513 on the promoter activity^10,22,52,53^. In a study by Osawa *et al*., carried out in insect cells, an increase on in the promoter activity was observed when replacing the C allele of rs1862513 by G^22^. The effect was particularly striking after co-transfection with SP1/SP3. An impact of this SNP was also evident in reporter assays o carried out in 3T3 fibroblast/adipocyte cells^10,52,53^ and in the monocytic cell line THP1^52^. However, in the latter study, instead of a single SNP, the rs34861192-rs1862513 haplotypes A-G and G-C were compared leaving it open which of these two SNPs are actually exhibiting the effect^52^. Thus, it cannot be excluded that rs1862513 may exhibit some cell-type specific effect.

An interacting mechanisms of CpG methylation and histone modifications in transcription regulation is well established^30,54^. A potential regulatory region has been proposed in the vicinity of rs34861192 which is the most methylated CpG within RETN promoter analyzed^16^. Therefore, the levels of methylation at CpG SNP within regulatory region can impact more widely the gene around the methylation site which could be also observed in the context of genetic regulation of Resistin when haplotypes based promoter methylation was compared. NF-kB family of transcription factors could inhibit sp1/sp3 mediated transcription as well as change the chromatin dynamics through recruitment of histone deacetylases (HDACs)^16,27,47,55–57^. This could also be applied in the context of p50/p50 mediated suppression of resistin transcription due to the close proximity of identified sp1/sp3 binding sites required for constitutive resistin transcription and a haplotype dependent acetylation of H3K27 at RETN gene locus.

The allelic states of the two RETN SNPs are indicative for the basal RETN expression in monocytes as well as for the RETN protein in the blood. High serum levels reportedly associate with T2D, coronary artery disease (CAD) and inflammatory diseases^1,3,5,33–35^. Although in GWAS studies the two RETN SNPs have not been found yet to be directly linked to these diseases^58–60^, they have been associated already with rheumatoid arthritis^61^ and cerebral infarction^62^. Moreover, in individuals of Chinese ethnicity, where the two SNPs are the predominant variants regulating the gene expression, the A-A haplotype (associated with high RETN levels) seems to promote the elimination of pathogens such as hepatitis C virus^63^.

In line with its established role in inflammation, the correlation of the RETN genotype with the plasma protein data of our cohort revealed significant associations with a number of immune mediators. Based on the IPA analysis the closest link was detected for the HMGB1, a pathway well-known to be associated with inflammatory diseases, T2D and CAD^37,38,64–66^. Notably, enhanced expression of both HMGB1 and RETN was reported in sepsis^6^. In this condition an unexpected protective role for RETN was identified, in which RETN promotes anti-inflammatory signaling and physically blocks the LPS binding to Toll-like receptor 4 (TLR4)^67^. In sepsis, increased levels were also detected for sRAGE, a soluble receptor variant blocking HMGB1/RAGE signaling^67–70^. In line with anti-inflammatory role RETN plays in this condition, sRAGE was positively associated with the high expressing A-A haplotype, while pro-inflammatory molecules S100B, IL-8, sTNFRI, and sTNFRII were inversely associated (Table 2).

Of the two haplotype variants, the constitutively repressed G-G haplotype represents the ancestral haplotype. The ‘dysregulated’ A-A haplotype, which has lost the transcriptional control by p50/p50, has been formed later and is found only in South Asian and some African populations as a minor variant (maf < 0.2) (Supplementary Table 6). The two G → A mutations causing the formation of rs34861192-rs3219175 A-A haplotype resulted in the loss of two linked repressor mechanisms, the p50/p50 homodimer binding and the promoter C-methylation, but it is unknown, why the de-repression occurred specifically in these populations. It might be caused by ethnic preferences in nutrition, the type of pathogen-exposure and other environmental peculiarities of the habitats requiring more effective pathogen-defense.

In conclusion we demonstrated in this study that the genetic regulation of RETN expression in human monocytes is based on two tightly linked SNPs controlling p50/p50 homodimer binding, histone acetylation and the C-methylation of the RETN promoter. The two RETN haplotypes formed by these SNPs may have evolved to balance improved pathogen defense with the inherent risk for inflammatory diseases in East Asian populations. Further in-depth study of the p50/p50 regulation are needed to fully understand the role of the NF-kB pathways in the suppression of this gene.

## Materials and Methods

### Cohorts

Most eQTL data and associated plasma- and blood cell-parameters were taken from a prior study of 202 healthy Singaporean individuals of Chinese ethnicity. Details on all cohorts had been published previously^17,18,20^. For additional functional experiments on monocytes, blood was taken from healthy donors of ethnic Chinese ethnicity. They were part of the Singapore Chinese Cohort Study, which was approved by the Institutional Review Board at the National University Hospital (IRB No. NUS 07-023, NUS 10-445 and NUS 09-256) and is in compliance with the Helsinki declaration. Written informed consent was obtained from all volunteers prior to the collection of blood samples.

### Genotyping

The rs34861192 and rs3219175 genotype of the healthy volunteers was determined using High Resolution Melting (HRM) analysis. DNA was isolated from whole blood or PBMCs using DNeasy Blood & Tissue Kits (Qiagen) according to the manufacturer’s instructions. The HRM analysis was performed by real-time PCR using a CFX96 Real-Time Detection System (Bio-Rad). The primers for rs3219175 genotyping were 5′-TCCAGCCCTTACTGTCTGCT-3′ (forward), 5′-ATCCGGGGCCAAGAGGAAGC-3′ (reverse) and for rs34861192 genotyping were 5′-TGCTGTGATCATAAGTCACTGTAG-3′ (forward), 5′-TGACGTGAGAGAATTGCTTGA-3′ (reverse); amplification was carried out using the following protocol: 3 min at 95 °C, 40 cycles of 5 sec at 95 °C and 5 sec at 50 °C, and final extension for 10 sec at 95 °C. A melting curve was generated from 65 to 95 °C (in 0.2 °C increments) with 10 sec/step. Heterozygosity and homozygosity of the allelic state were deduced using Precision Melting Analysis software (Bio-Rad).

### RNA extraction and qPCR analysis of monocytes

Total mRNA from monocytes was isolated using the RNeasy Mini Kit (Qiagen) and Superscript™ II Reverse Transcriptase (Invitrogen; 18064-14) with hexamer primer (Roche Diagnostics). A real-time PCR was performed with 10 ng of cDNA and oligonucleotide primers (300 nmol/L) and probes (100 nmol/L) in the CFX96 real time system (Bio-Rad). Primers and probes (RETN; 4351372, GAPDH; 4331182) were from Thermo Fisher Scientific. All TaqMan reagents were obtained from Thermo Fisher Scientific. The following PCR conditions were used for the LightCycler: 2 min, 50 °C, and 10 min, 95 °C, followed by 40 cycles of 15 s, 95 °C and 1 min, 60 °C in 20 ul reactions.

### eQTL analysis

Whole blood gene expression data^17,19^ was processed using the Bioconductor lumi package in R 3.1.2. The gene expression data was quantile-normalized and log2 transformed prior to further analysis. eQTL analysis was done using MatrixEQTL using a cis distance of 100,000 bp on 206 subjects with 2,612,710 quality controlled SNPs. Conditional SNP association analyses were also conducted using PLINK conditional on the SNP rs3219175. The beta value, test value (as stat) and test p-value are reported in the relevant table.

### Monocyte isolation

Monocytes used for nuclear protein extraction were isolated from apheresis blood provided by the blood bank of the Health Sciences Authority (HSA), Singapore. Monocytes used for mRNA-, ChIP- and bisulfite sequencing analysis were isolated from PBMCs derived from healthy volunteers by Magnetic cell sorting using MACS Human CD14 Microbeads (Miltenyi Biotec) according to the instructions recommended by manufacturer.

### Nuclear protein isolation

Monocytes were washed with ice-cold PBS, followed by buffer A (20 mM HEPES, pH 7.9, 20% Glycerol, 10 mM NaCl, 0.2 mM EDTA (pH 8), 1 mM DTT, 0.1% Triton X-100) supplemented with Pierce Proteases and Phosphatase Inhibitor Mini Tablets (Thermo Fisher Scientific). After 15 min incubation on ice, homogenates were centrifuged at 4 °C for 15 min at 2,000 rpm, and the resultant nuclear pellets were re-suspended in buffer A containing 500 mM NaCl. The nuclear proteins were incubated for 1 h on ice with intermittent tapping followed by 15 min centrifugation at 13,000 rpm (4 °C). The supernatants were then aliquoted and snap-frozen and stored at −80 °C until used. Quantitation of nuclear proteins was performed using Bio-Rad protein quantitation kit (Bradford Assay).

### Electrophoretic mobility shift assay (EMSA)

Allele-specific oligonucleotides corresponding to the RETN promoter region were designed with the allelic G or A base pairs of rs3219175 and rs34861192 located in the center of the respective probe (termed as ‘G probe’ and ‘A probe’). The double-strand oligonucleotides had a length of 25 bp; the sequences are listed in Supplementary File 4. G- and A-probe oligonucleotides were synthesized by IDT, Singapore; all remaining oligonucleotides were purchased from Santa Cruz Biotechnology. ^32^P labelling was introduced using T4 Polynucleotide Kinase. For EMSA reactions, 5 μg of the monocyte nuclear protein extract was incubated with 40,000 cpm of the ^32^P-labeled oligonucleotide probe in a total volume of 20 μl 5 mM HEPES (pH 7.9), 5% glycerol, 0.5 mM EDTA, 1 μg dIdC, 1 mM DTT, and 80 mM NaCl at 4 °C for 45 min. For ‘cold’ competition experiments, 12.5–50 ng of the unlabeled oligonucleotide competitor were added 15 min before adding the respective radiolabeled probe (representing a 12.5–50 fold excess of the unlabeled competitor). For supershift assays, 2 ul of either rabbit polyclonal α-p50 IgG (H-135 X, Santa Cruz Biotechnology), α-p52 IgG (H-135 X, Santa Cruz Biotechnology), α-p65 IgG (H-135 X, Santa Cruz Biotechnology), α-c-REL IgG (H-135 X, Santa Cruz Biotechnology), α-Rel B IgG (H-135 X, Santa Cruz Biotechnology), α-p50 IgG (H-135 X, Santa Cruz Biotechnology) or rabbit IgG (Abcam) were added 15 min before adding the respective radiolabeled probe. After incubation, the DNA–protein complexes were separated for 90 min with a 5% non-denaturing polyacrylamide gel at 240 V on a protein II gel-apparatus (Bio-Rad) using 0.5X Tris/Borate/EDTA (TBE). After electrophoresis, the gel was transferred to a Whatman paper and dried on a vacuum gel dryer (Gel Dryer 583, Bio-Rad) for 2 h at 80 °C. The dried gel was then exposed on an imaging plate and scanned using a Fuji FLA-5000 phospho-image scanner.

### Transient transfection and luciferase assay

The human resistin promoter segment was PCR-amplified using genomic templet DNA isolated from genotyped donors. The fragments were then cloned in into the PGL3 basic plasmid using Mlu I and Bgl II restriction sites (Supplementary File 3). For transfection, U937 cells were maintained in RPMI 1640 media with 10% fetal calf serum and antibiotics. Exponentially growing cells were harvested and resuspended at a density of 2 × 10^7^/ml in RPMI media without FCS supplemented with 10 mM Dextrose and 0.1 mM DTT. 0.3 ml of the cell suspension was used for per electroporation in 0.4 cm cuvettes. For electroporation, 10 ug of reporter Plasmid and 1 ug of renilla luciferase (10:1) were mixed with 0.3 ml of cell suspension and electroporated using Bio-Rad Gene pulser II instrument (220 V and 960 uF capacitance). 48 h post electroporation, cells were lysed in 1x PLB and analyzed for luciferase and renilla activity using the Dual-Glow luciferase assay kit GloMax® Luminomete (Promega Corporation).

### Confocal microscopy

Monocyte purity of cell preparations used for confocal microscopy was >90% (based on flow cytometry analysis). The cells were deposited onto the glass slide by centrifugation at 400 rpm for 5 min with Shandon Cytospin 4 cytocentrifuge. The cells were then fixed with 4% paraformaldehyde (PFA), incubated for 15 min at room temperature, followed by 3 times washing with PBS and incubated for 2 h in blocking buffer (5% FBS, 0.3% Triton X™-100 in PBS). The cells were then stained overnight at 4 °C with anti-NFκB p65 Antibody (1:50)(#sc-8008, Santa Cruz) and anti-NFκB p50 Antibody (NLS) (1:50)(#sc-114, Santa Cruz) in antibody dilution buffer (1% BSA, 0.3% Triton X™-100 in PBS). Cells were washed 3 times with PBS and then stained with goat anti-rabbit IgG Alexa Fluor 633 (#A21070, ThermoFisher Scientific) and goat anti-mouse Alexa Fluor 488 (#A11001, ThermoFisher Scientific). Cells were washed 3 times with PBS and stained for 15 min with Hoechst 33342 (1:10,000) (#H3570, ThermoFisher Scientific). Cells were mounted using MOWIOL® 4–88 Reagent (#475904, EMD Millipore) before viewing by confocal microscopy (Olympus).

### NFKB1/p50 inhibition assay

Freshly isolated monocytes from individuals genotyped for rs3219175 were treated for 24 h with 30 µM of either p50-specific cell permeable peptide inhibitor (NFkB-SN50; Merck Millipore) or control peptide. Post treatment RNA was isolated, reverse transcribed and analyzed for mRNA expression of RETN.

### Chromatin Immunoprecipitation assay (ChIP-Assay)

Chromatin immunoprecipitation (ChIP) assays were performed on monocytes isolated from three AG- and three GG-donors (rs3219175). ChIP was carried out essentially as previously described [16]. Briefly, DNA/protein cross-linking was achieved by incubating the cells (freshly isolated monocytes; 5 × 10^6^ in10 ml of PBS) for 10 min at 37 °C in 1% formaldehyde. Ultrasound sonication of the chromatin was performed in lysis buffer (50 mM Tris-Cl pH 8.0, 10 mM EDTA, 1% SDS) with 3 min cycles [30 sec “ON”, 30 sec “OFF”] for a total of 10 cycles using a Bioruptor UCD-300 sonicator (Diagenode). After sonication, samples were diluted in 5-fold dilution buffer (0.01% SDS,1.1% Triton X-100, 1.1 mM EDTA, 20 mM Tris-Cl pH 8.0) followed by overnight incubation at 4 °C with 5 µg of either rabbit polyclonal anti-p50 antibody IgG (SC-114, Santa Cruz Biotechnology), or rabbit polyclonal IgG (SC-2027, Santa Cruz Biotechnology). Real-time PCRs of genomic regions containing the putative p50-binding site was performed in triplicate by using iTaq SYBR green supermix (Bio-Rad) with RETN promoter specific primers (forward primer: 5′-CTGTTGGAAGTGGGAAGGCTC-3′; reverse primer: 5′-CTGGCTTGGCTAATAAGTCCCTG-3′). The relative occupancy of the immunoprecipitated factor at RETN locus is estimated by using the comparative threshold method [16]. 2^(Ctmock–Ctspecific)^, where Ct_mock_ and Ct_specific_ are mean threshold cycles of PCR done in triplicate on DNA samples from mock and specific immunoprecipitation.

### CRISPR/Cas9 mediated knock out

To generate a p50 knock out in U937 cells, a pair of guide RNAs targeting exon1 of NFKB1 was synthesized (IDT Singapore) and cloned into pLenti-CMV-CAS9-T2A-GFP) plasmid. The sequence of the guide RNA was CAGGTAGTCCACCATGGGAT and GAACAAGAAGTCTTACCCTC. U937 cells were infected with virus particles containing either guide RNAs targeting NFKB1 or nan-targeting gRNAs as negative control. Single cell sorting into 96 well plates was performed 48 h post infection and the clones expanded for two weeks. Clones were then tested by PCR amplification of targeted genomic region (Forward: TGGCAGCAGCAATTTAAGACAAG Reverse: GGGTACTTTCAGGCTCTCTATGG) followed by Sanger sequencing (AIT Singapore). In selected clones, which exhibited the desired genetic defect, the gene knock out was validated at the protein level by western blot (Clones 2 and 4 were used for experiments) (Supplementary Fig. 6).

### Bisulfite treatment and DNA methylation analysis

Primers for the bisulfite genomic sequencing (BGS) analysis were designed to cover the RETN promoter region 301–771 bp (470 bp) upstream to the transcription start site of RETN as well as a 495 bp segment of the RETN 3′-UTR (Supplementary Fig. 7). DNA bisulphite treatment of the genomic DNA was performed as described before^17^ according to the protocol provided with the EZ DNA Methylation-Direct™ Kit (Zymo Research). For BGS, bisulfite-treated DNA was amplified using the RETN-specific primers (RETN promoter: forward primer 5′-TGGGTATTTGGGTATGAATGTGGTAT-3′, reverse primer 5′-TAATAAATCCCTAAACCCCCAACCC-3′; RETN 3′-UTR: forward primer 5′-TTTTTGTGTTTCGGGTTGTAGGTTT-3′, reverse primer 5′-ACCCTATTTTCGAAAAAAACAATTAAAAACCC-3′). The PCR products were cloned into the TA cloning vector (Invitrogen, Life technologies) and minimum four and maximum five clones from each donor were sequenced using M13 forward primer. Bis-sulphite DNA-sequenced clones from genotyped monocytes were analyzed using APE plasmid editor by aligning of sequencing data with reference (Supplementary Fig. 7). This allowed to define the methylation state at each CpG sites within RETN promoter and 3′-UTR through binary analysis. The percentage of methylation was computed for each donor at each available CpG sites as the fraction of the clones which were methylation positive over all the clones (Supplementary Table 5). The promoter methylation percentage was computed as the average of the percentage of methylation at all available methylation CpG sites for each donor. Kruskal-Wallis test was used for statistical analysis of the promoter methylation percentage.

### Monocytes specific haQTL analysis

The complete haQTL analysis will be published elsewhere (Poschmann & del Rosario *et al*.; in submission; Genome wide data has been deposited at the European Genome-phenome Archive EGA, http://www.ebi.ac.uk/ega/), which is hosted by the EBI, under accession number EGAS00001002997.). Detection of haQTLs was performed as described in^31^. Briefly, H3K27ac ChIP-seq was performed on CD14^+^ monocytes isolated using CD14^+^ immunomagnetic separation beads after isolation from the 32 individuals (MACS, Miltenyi). After mapping, normalization and peak calling, we used the G-SCI test to call histone acetylation QTLs^31^. The G-SCI test detects SNPs whose genotypes correlate with ChiP-seq peak heights without requiring hard genotypes and is a sensitive test due to its use of both peak heights and allelic imbalance on the reads mapping on the SNP. haQTL *P*-values were computed using permutation followed by FDR correction and filtering for effect size as done before^31^.

### Flow cytometry analysis

Blood samples were collected in either BD K2EDTA or citrate vacutainer tubes. FACS staining was performed on either whole blood samples or isolated PBMCs^18^. Erythrocytes were removed from whole blood by incubation in 14 mL erythrocyte lysis buffer (155 mM NH4Cl, 10 mM KHCO3, and 0.1 mM EDTA) for 10 min at room temperature. After erythrocytes lysis, the cells were centrifuged at 860 × g for 3 min. The cell pellets were re-suspended in PBS and washed once with PBS by centrifugation at 860 × g for 3 min at room temperature. For PBMC isolation, whole blood was layered on Ficoll-Paque (GE Healthcare) and centrifuged for 500 × g for 30 min without breaking. PBMCs were harvested at the interface between Ficoll and plasma layers. To discriminate live from the dead cells, cells were incubated with 100 μL of LIVE/DEAD Fixable Aqua Dead kit (Life Technologies) in PBS for 10 min at room temperature. The cells were washed once with 100 μL MACS buffer (0.5% BSA, 2 mM EDTA in PBS) and transferred into 96 V-bottom plates for centrifugation at 1000 × g for 3 min. For immune subsets analysis, cells were stained with a cocktail of 7 antibodies: anti-FCERI (AER-37), anti-CD123 (6H6) anti-CD14 (61D3), anti-CD16 (3G8), anti-HLA-DR (L243), anti-CD1c (L161), and anti-IgE (MB10-5C4) mAb. Stained cells were determined using a LSRII or a Fortessa flow cytometer (BD Biosciences). Gating is shown in Supplementary Fig. 9.

### Analysis of plasma components

Plasma samples were obtained by centrifugation of whole blood collected in BD 8 ml sodium citrate, mononuclear cell preparation tubes (CPT) according to manufacturer’s instructions. All plasma components were quantified by Luminex as described earlier^18^. PLINK (version 1.90b3.46) was used to analyze the SNP association with the logarithmically transformed protein measurements. The beta value, Wald test value (as stat) and Wald test p-value are reported in the relevant table.

### Statistical analysis

Data were considered non-parametric unless otherwise stated and therefore non-parametric statistical comparisons were performed using Prism 6 software (GraphPad Software, Inc., La Jolla, CA, USA).

## Supplementary information

Supplementry info
