# Supplementary material for: Resistin expression in human monocytes is controlled by two linked promoter SNPs mediating NFKB p50/p50 binding and C-methylation
Source: Sci Rep. 2019 Oct 23;9:15245. doi: 10.1038/s41598-019-51592-0 (PMC6811637; doi:10.1038/s41598-019-51592-0)
Supplement: Supplementary file 1 — Supplementry info [file 41598_2019_51592_MOESM1_ESM.docx]

**Resistin expression in human monocytes is controlled by two linked promoter SNPs mediating NFKB p50/p50 binding and C-methylation**

Dilip Kumar^1^, Bernett Lee^1^, Kia Joo Puan^1^, Wendy Lee^1^, Boris San Luis^1^, Nurhashikin Yusof^1^, Anand Kumar Andiappan^1^, Ricardo Del Rosario ^3, 5^ Jeremie Poschmann^3,6^, Pavanish Kumar^1^, Gennaro DeLibero^1, 4^, Amit Singhal^1^, Shyam Prabhakar^3^, Wang De Yun^2^, Michael Poidinger^1^, Olaf Rötzschke^1^

^1^Singapore Immunology Network (SIgN), A*STAR (Agency for Science, Technology and Research), Singapore; ^2^Department of Otolaryngology, National University of Singapore; ^3^Genome Institute of Singapore (GIS), Agency for Science, Technology and Research of Singapore (A*STAR), Singapore; ^4^ Department of Biomedicine, University of Basel, Basel, Switzerland; ^5^Stanley Center for Psychiatric Research, Broad Institute of MIT and Harvard, 75 Ames St., Cambridge MA 02142, USA; ^6^Centre de Recherche en Transplantation et Immunologie, Université de Nantes, France.

Corresponding authors:

Olaf Rötzschke, Ph.D.

Singapore Immunology Network (SIgN)

8A Biomedical Grove #04-06, Singapore 138648

olaf_rotzschke@immunol.a-star.edu.sg

Dilip Kumar, Ph.D.

Singapore Immunology Network (SIgN)

8A Biomedical Grove #04-06, Singapore 138648

dilip_kumar@immunol.a-star.edu.sg

**Supplementary tables**

**Supplementary figures**


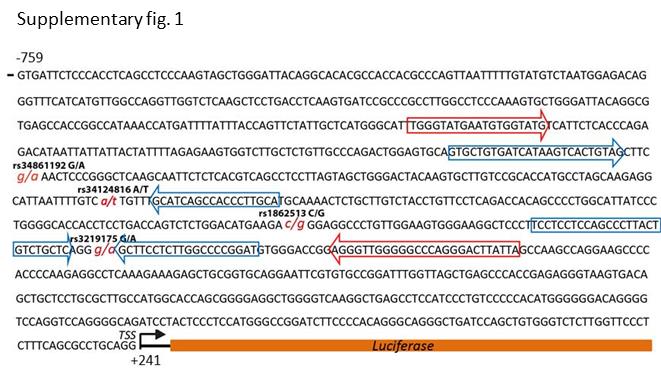


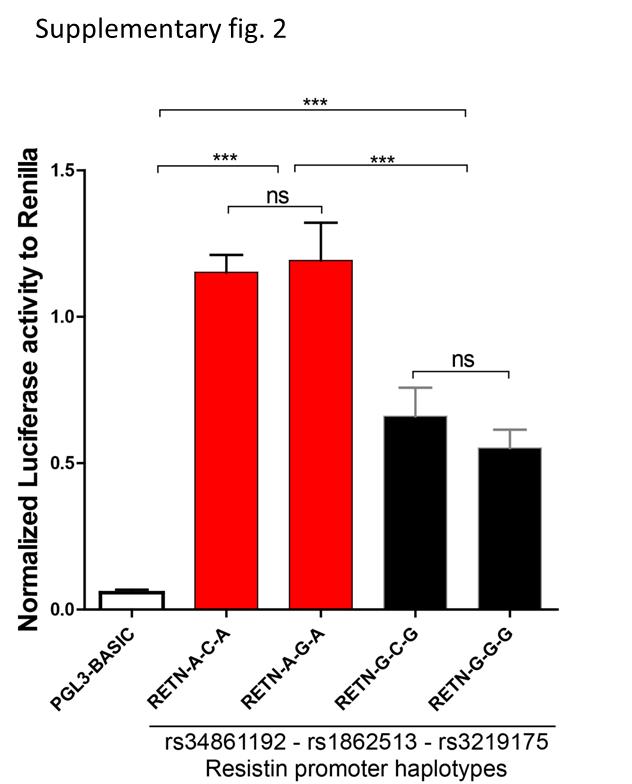


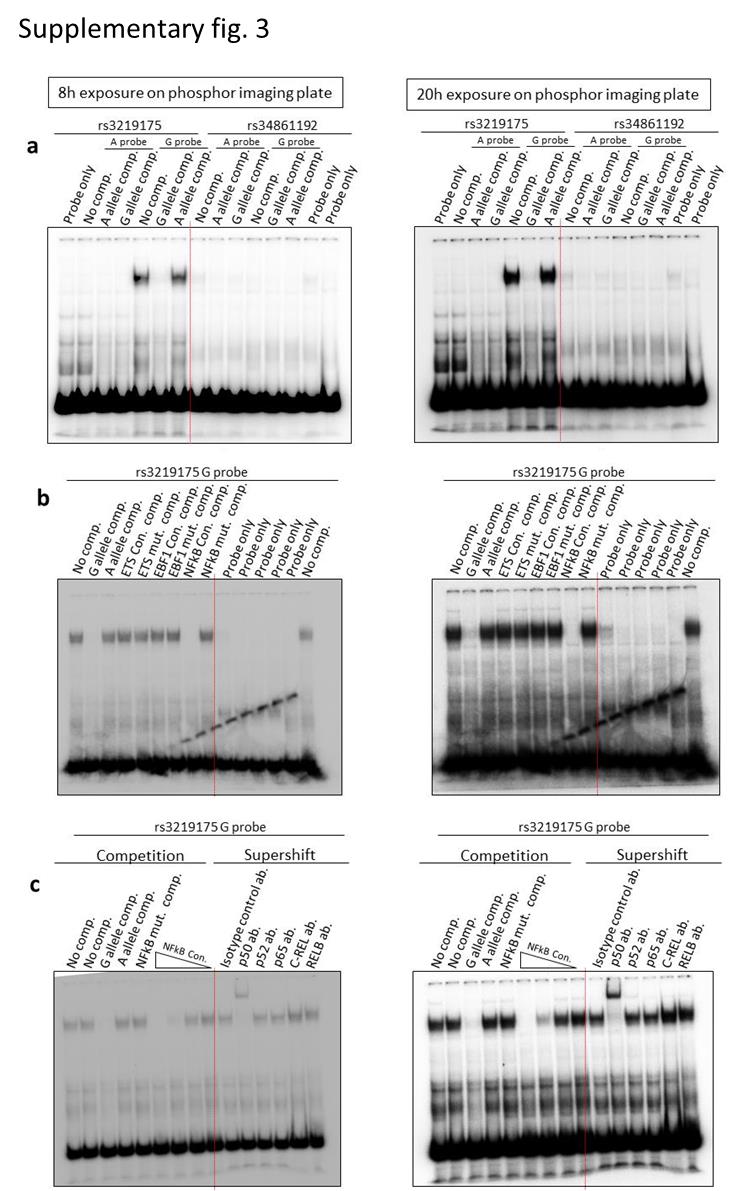

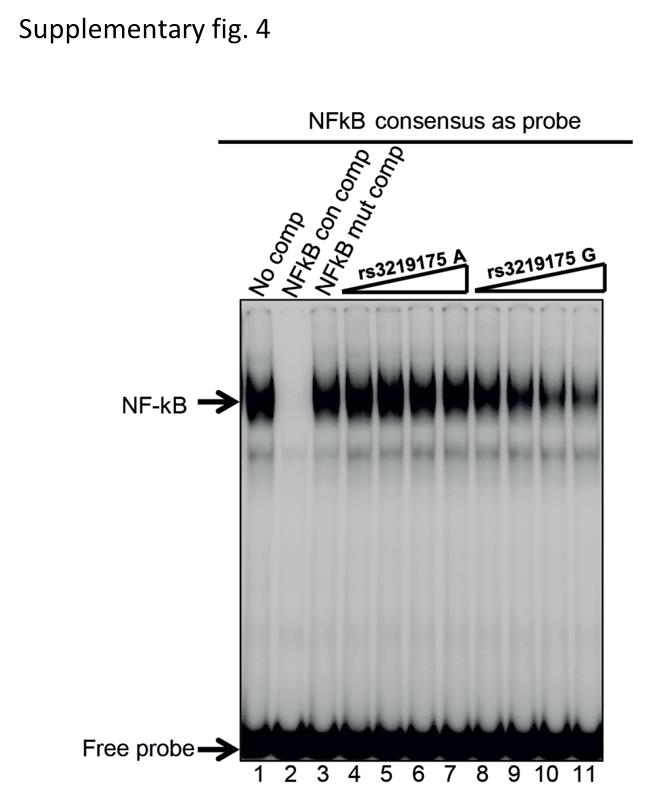

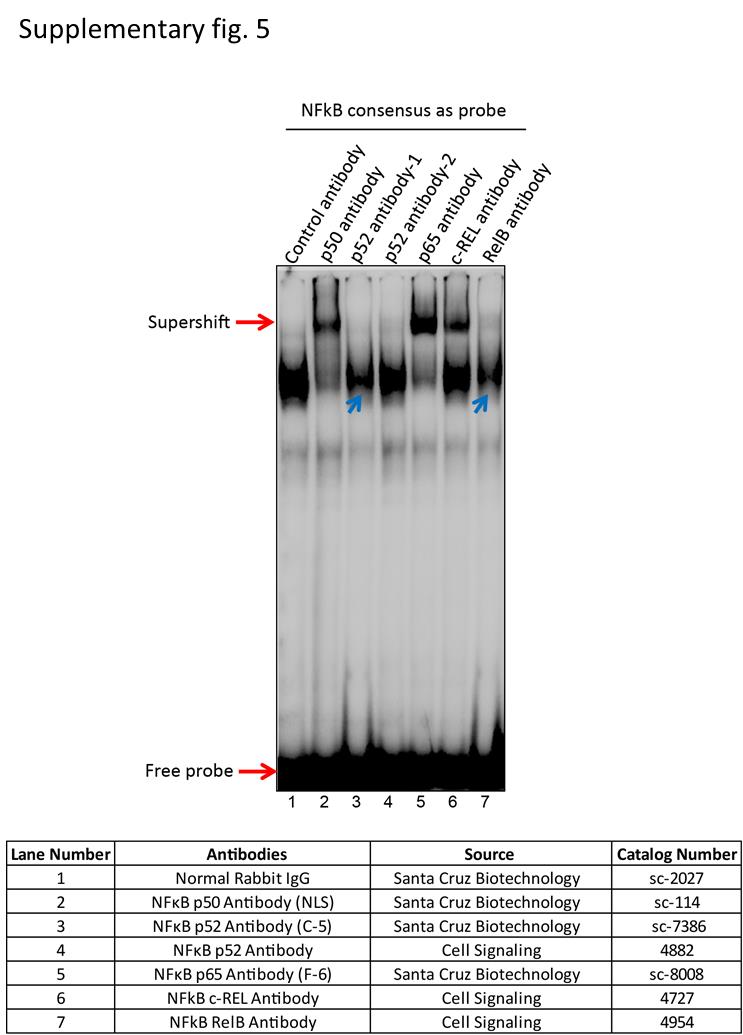


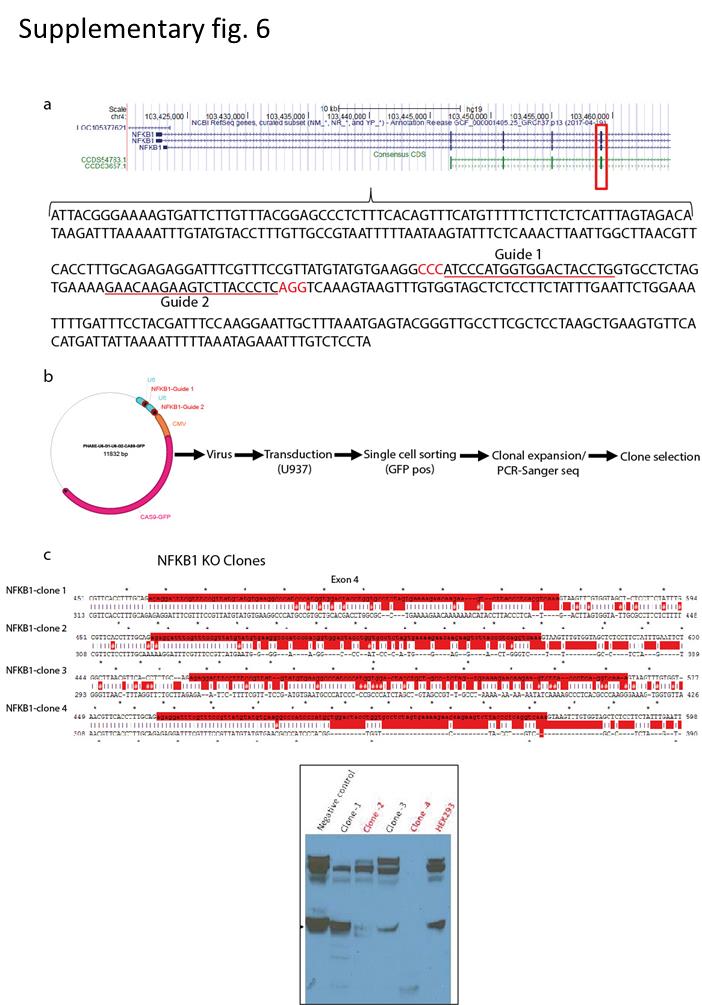

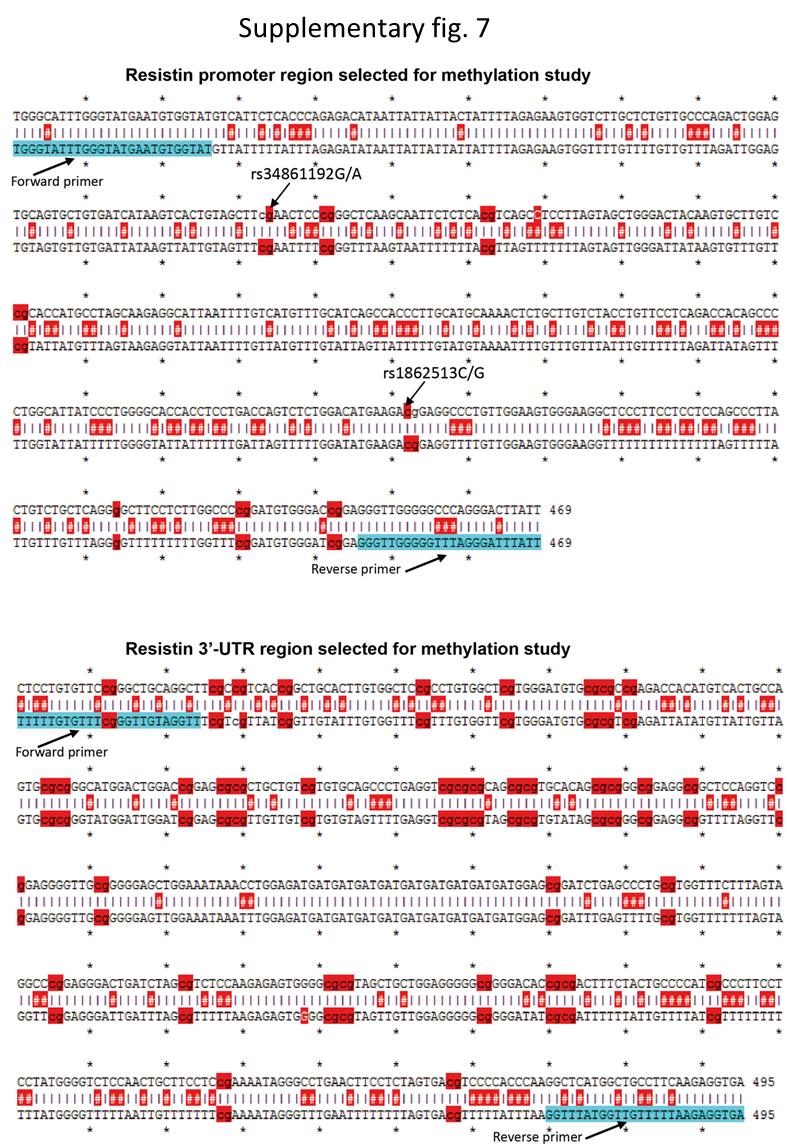


Supplementary Fig. 8


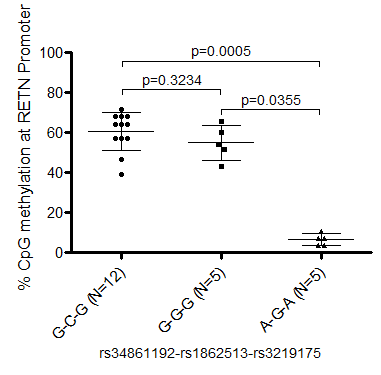


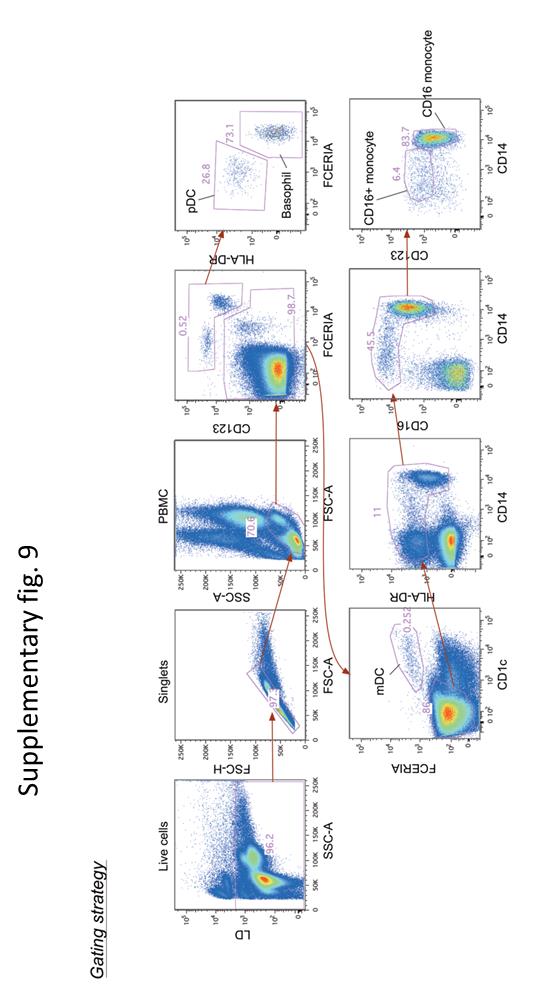


**Legend to Supplementary tables and figures**

**Supplementary table 1: Conditional SNP-SNP interaction analysis**

Conditional SNP association analyses were conducted SNPs with FDR < 0.05 using PLINK conditional on the SNP rs3219175. The genomic position within chromosome 19, allele 1 (minor allele), the number of samples (n), regression coefficient, standard error, t statistic and the nominal p values are reported in the table.

**Supplementary table 2: RETN associated SNPs in monocyte eQTLs**

Monocyte specific genome-wide eQTLs for Asian populations have been published by Raj.et.al (21). RETN specific eQTLs for monocytes were extracted from the data provided by the authors

**Supplementary table 3: Oligonucleotides for probes used in EMSA experiments.**

The table lists the sequence of oligonucleotides used as radiolabelled probes or as cold competitors in EMSA. RETN promoter specific probe sequences represent the two allelic variants of a 25 bp region containing the rs3219175G/A, rs34861192G/A SNP. Consensus and mutant oligonucleotides are based on consensus binding sites of the respective transcription factor. They were purchased either as predesigned versions directly from the manufacturer or were custom made based on a previous publication

**Supplementary table 4: TRANSFACT analysis of rs3219175G/A**

In silico prediction of transcription factors with allele-specific binding to rs3219175G/A. The web based prediction tool TRANSFAC was used to identify potential transcription factor binding sites in RETN promoter affected by the allelic variations of rs3219175G/A. Only sites predicted with a binding score greater than core score >0.9 and full score >0.8 were selected for further analysis. Yellow shaded rows indicate transcription factors highly specific for G allele.

**Supplementary table 5: Summary of the methylation status at CpG sites within RETN promoter**

Multiple clones (total 93) were sequenced for 22 donors (12 Donors: G-C-G, 5 Donors: G-G-G and 5 Donors: A-G-A) covering all three major haplotypes G-C-G, G-G-G and A-G-A, sequencing data was used to compute percentage of methylation at each available CpG site for each donor as the fraction of the clones which were methylation positive over all the clones. Average of RETN promoter methylation was computed using average methylation at each available CpG site within the analysed promoter region in all three haplotypes. Haplotype^a^ represents the status of the allelic states at rs34861192-rs1862513-rs3219175; the “0^b^” in the columns indicates that the CpG methylation site is lost due to the change in the allelic state of the SNP. Two of the CpG sites, rs34861192 and rs1862513 were thus affected and therefore excluded from the averaged promoter methylation values.

**Supplementary table 6: Allelic distribution of RETN associated SNPs**

Allele frequencies of rs34861192 in three major ethnical groups. The table displays the frequencies of the G-allele and the T-allele for Caucasian, Han Chinese and Japanese based on 1000 Genome project and Hap Map.

**Supplementary fig 1: RETN promoter sequence used for reporter assays**

The regions 1kB bases upstream of the transcription start site (TSS) (https://genome.ucsc.edu/) of two donors were cloned into the PGL-basic reporter vector. The donors differed in the allelic states of three SNPs rs3219175G/A, rs34861192G/A and rs1862513C/G. The location of is indicated (italic, small, red and underlined). The transcription start site is indicated by ‘TSS’ followed by luciferase gene. The primer sets for genotyping are indicated by blue arrows and primers for bisulphite assisted sequencing red arrows.

**Supplementary fig. 2: Reporter assay of modified RETN promoter**

In order to determine the influence of rs1862513 on the RETN expression its allelic state in the two RETN promoter fragments was swapped by CRISPR. Four luciferase reporter vectors containing the G-C-G, G-G-G, A-G-A and A-C-A haplotype (rs34861192-rs1862513-rs3219175) and the pGL3-basic vector were co-transfected with pTK-RL (internal control vector harbouring renilla luciferase gene) into U937 cells. The bar chart indicates the firefly luminescence normalized by renilla luminescence.

**Supplementary fig. 3: Original EMSA competition and Super-shift data**

Section a, b and c of figure 2 was generated based on the EMSA competition and super-shift experimental data shown here. Super-shift assays were carried out with the radiolabelled NFkB consensus probe. Specificity and source of the antibodies against members of the NFkB family are given in table below. Super-shifted bands and bands lost by the antibody treatment are indicated by red and blue arrows, respectively. Validated antibodies used for the super-shift experiments (Fig 2c) are p50 (sc-114), p52 (sc-7386), p65 (sc-8008), c-REL (cs-4727), and RELB (cs-4954).

**Supplementary fig. 4: EMSA competition of NFkB family-consensus probes**

EMSA competition of radiolabelled NFkB consensus probes with unlabelled G and A probes of rs34861192 are shown. Nuclear extracts from primary monocytes were exposed to radio-labelled NFkB consensus alone (lane 1) or together with increasing amounts of unlabelled A probe (lane 4 – 7) or G probe of rs34861192G (lane 8 – 11). Competition with an excess of unlabeled NFkB consensus (lane 2), or a mutated version (lane 3) was carried out as control.

**Supplementary fig. 5: Validation of NFkB antibodies for super-shift assays**

Super-shift assays were carried out with the radiolabelled NFkB consensus probe. Specificity and source of the antibodies against members of the NFkB family are given in table below. Super-shifted bands and bands lost by the antibody treatment are indicated by red and blue arrows, respectively. Validated antibodies used for the super-shift experiments (Fig 2c) are p50 (sc-114), p52 (sc-7386), p65 (sc-8008), c-REL (cs-4727), and RELB (cs-4954).

**Supplementary fig. 6: Generation of NFKB1 knock out cell lines**

(a) Paired guide RNAs (PAM sequences in red) were cloned in a lentiviral vector containing CAS9-GFP, targeting the first exon of NFKB1 gene (b) The virus was produced by co-transfection of packaging and paired gRNAs-CAS9-GFP in HEK293 cells, followed by U937 cell transduction and single cell sorting (c). Expanded single cells clones were for further for the editing at genomic level (Exon 4) by PCR amplification of targeted regions and Sanger sequencing. Loss of protein level in all four selected clones were verified by western blot, clone 2 and 4 showed the loss of protein level due the editing within exon 1 of NFKB1 gene and were used for experiments described in fig. 2g

**Supplementary fig. 7: RETN promoter and 3’-UTR regions for methylation studies**

(a) The figure shows a representative example of a bisulfite conversion of the promoter region. The unconverted (upper sequence) and bis-converted (lower sequence) RETN promoter fragment was aligned for the detection of C-methylation. All CpG-SNPs are indicated by arrows, PCR amplification primers and CpG are highlighted in light blue and red, respectively. (b) Alignment of unconverted (upper sequence) and bis-converted (lower sequence) RETN 3’-UTR fragments.

**Supplementary fig. 8: CpG SNP rs1862513 has no significant impact at RETN promoter methylation**

The figure is based on the data presented in supplementary table 5. It was generated using 93 T-A clones from 22 donors and displays the average percentage of methylation on all available CpG sites of the RETN promotor for the three haplotypes G-C-G, G-G-G and A-G-A (rs34861192-rs1862513-rs3219175). No significant difference in average promoter methylation was observed between G-C-G and G-G-G, while significant differences exist between G-C-G vs A-G-A and G-G-G vs A-G-A. Kruskal-Wallis test was performed for statistical analysis

**Supplementary fig. 9: Gating strategy for the FACS-analysis for various immune cells**

RBC-lysed whole blood samples were stained with LIVE/DEAD Fixable Aqua Viability Dye followed by a cocktail of 7 antibodies including anti-FCERI (AER-37), anti-CD123 (6H6) anti-CD14 (61D3), anti-CD16 (3G8), anti-HLA-DR (L243), anti-CD1c (L161), and anti-IgE (MB10-5C4) mAb. Typically, a total of 300,000 - 400,000 events were acquired for each sample. Gating shows the stepwise separation of PBMC, mDC and monocytes respectively. To analyze the percentage of mDC and monocytes to PBMC, first lymphocyte cellular fractions from whole were gated out followed by mDC and monocytes using their respective cell surface markers. mDC and monocytes shown here are positive for FCERI, CD1c, CD123, HLA-DR and CD14 respectively.
